# Rational and design of an individual participant data meta-analysis of spinal manipulative therapy for chronic low back pain—a protocol

**DOI:** 10.1186/s13643-017-0413-y

**Published:** 2017-01-26

**Authors:** A. de Zoete, M. R. de Boer, M. W. van Tulder, S. M. Rubinstein, M. Underwood, J. A. Hayden, J. Kalter, R. Ostelo

**Affiliations:** 1 Department of Epidemiology and Biostatistics, EMGO+ Institute for Health and Care Research, VU University Medical Center, Amsterdam, Netherlands; 2 Department of Health Science, Institute for Health and Care Research, Faculty of Earth & Life Science, VU University, De Boelelaan 1085, 1081HV Amsterdam, The Netherlands; 3 Warwick Clinical Trials Unit, Warwick Medical School, The University of Warwick, Coventry, CV4 7AL UK; 4 Department of Community Health & Epidemiology, Dalhousie University, Halifax, Nova Scotia B3H 1V7 Canada

**Keywords:** Low back pain, Spinal manipulative therapy, Individual participant data

## Abstract

**Background:**

Chronic low back pain (LBP) is the leading cause of pain and disability, resulting in a major socioeconomic impact. The Cochrane Review which examined the effect of spinal manipulative therapy (SMT) for chronic LBP concluded that SMT is moderately effective, but was based on conventional meta-analysis of aggregate data. The use of individual participant data (IPD) from trials allows for a more precise estimate of the treatment effect and has the potential to identify moderators and/or mediators. The aim is (1) to assess the overall treatment effect of SMT for primary and secondary outcomes in adults with chronic LBP, (2) to determine possible moderation of baseline characteristics on treatment effect, (3) to identify characteristics of intervention (e.g., manipulation/mobilization) that influence the treatment effect, and (4) to identify mediators of treatment effects.

**Methods:**

All trials included in the Cochrane Review on SMT for chronic LBP will be included which were published after the year 2000, and the search will be updated. No restrictions will be placed on the type of comparison or size of the study. Primary outcomes are pain intensity and physical functioning. A dataset will be compiled consisting of individual trials and variables included according to a predefined coding scheme. Variables to be included are descriptive of characteristics of the study, treatment, comparison, participant characteristics, and outcomes at all follow-up periods. A one-stage approach with a mixed model technique based on the intention-to-treat principle will be used for the analysis. Subsequent analyses will focus on treatment effect moderators and mediators.

**Discussion:**

We will analyze IPD for LBP trials in which SMT is one of the interventions. IPD meta-analysis has been shown to be more reliable and valid than aggregate data meta-analysis, although this difference might also be attributed to the number of studies that can be used or the amount of data that can be utilized. Therefore, this project may identify important gaps in our knowledge with respect to prognostic factors of treatment effects.

**Systematic review registration:**

PROSPERO CRD42015025714

**Electronic supplementary material:**

The online version of this article (doi:10.1186/s13643-017-0413-y) contains supplementary material, which is available to authorized users.

## Introduction

### Background

Low back pain (LBP) is one of the leading causes of pain and disability and has a major socioeconomic impact [1, 2]. The majority of the costs associated with LBP are generated by participants whose condition proceeds to chronicity. There is evidence that the costs of chronic LBP are rising, while the prevalence remains the same [3]. Spinal manipulative therapy (SMT) is a commonly used strategy to treat chronic LBP and is one of the several interventions which evidence suggests is moderately effective [4].

SMT is defined as including both spinal manipulation and mobilization and is the experimental intervention examined in this review. Unless otherwise indicated, SMT refers to both of these “hands-on” treatments [5, 6]. Mobilizations use low-grade velocity, small or large amplitude passive movement techniques within the participant’s range of motion and control. Manipulation, on the other hand, uses a high-velocity impulse or thrust applied to a synovial joint over a short amplitude at or near the end of the passive or physiologic range of motion, which is often accompanied by an audible “crack” [7]. The cracking sound is caused by a cavitation of the joint, which is a term used to describe the formation and activity of bubbles within the fluid [8].

Many hypotheses exist regarding the mechanism of action for spinal manipulation and mobilization [9–11], and some have postulated that given their theoretically different mechanisms of action, mobilization and manipulation should be assessed as separate entities [8]. The modes of action might be roughly divided into mechanical and neurophysiologic. The mechanical approach proposes that SMT acts on a manipulable lesion (often called the functional spinal lesion or subluxation) to reduce internal mechanical stresses resulting in reduced symptoms [12]. However, given the non-nociceptive behavior of chronic LBP, a purely mechanical theory alone cannot explain clinical improvement [8].

The neurophysiologic mechanism proposes that SMT impacts the primary afferent neurons from the paraspinal tissues, the motor control system, and pain processing [10], although the actual mechanism remains debatable [8, 9].

In back pain research on the effect of SMT, identification of relevant patient subgroups is an important goal [13, 14]. For clinicians, a source of frustration in back pain research has been the lumping of patients together as “non-specific LBP” even though there is an underlying presumption that relevant subgroups of individuals with chronic LBP exist. There has been extensive international attention in this area, which aims to identify patient-level characteristics that modify treatment effect [15]. In addition to effect modification, it is important to identify and subsequently target critical intervention components (that is, mediators of intervention effect). Mediators are causal links between the intervention and the outcome and identify how an intervention might achieve its effects. However, evidence on this topic is scarce or lacking altogether. Still this presumption of relevant subgroups may be one of the reasons that results from studies so far have, at best, only shown modest average treatment effects. The resulting lack of this knowledge may hamper clinical decision-making in LBP by clinicians.

Another challenge in the investigation of treatments for LBP is to adequately assess the exact content and complexity of clinical management. Commonly, SMT are delivered as a “programme of care” rather than a specific individual treatment. We observed this in a previous work; SMT is often provided in combination with advices and/or exercises and it is difficult to separate the effect of the different components. Also, SMT intervention encompasses a heterogeneous group of interventions including different types of treatment (e.g., high-velocity low-amplitude (HVLA) manipulation versus low-velocity passive or resisted movement mobilization) [4]. Furthermore, most LBP trials are underpowered to detect modifiers of treatment response [16].

It is necessary to identify relevant differences in treatment and patient subgroups to get a better understanding of the “best” strategies for SMT in chronic LBP patients. This can potentially lead to better informed clinical practice.

### Individual participant data meta-analysis

In a traditional systematic review, published data are summarized in meta-analyses resulting in differences in mean treatment effect. This standard approach of pooling data increases statistical power and allows the effect sizes to be estimated with greater precision.

However, meta-analyses that collect published aggregate study data and pool study results have limitations. For example, subgroup data are typically not presented and the power to detect true effect modifiers is low [17]. Therefore, as some argue, meta-analyses that, bring together this heterogeneous information, have limited relevance in the management of individual patients in clinical practice [18].

An alternative approach to evidence synthesis is meta-analysis of individual participant data meta-analysis (IPD). IPD meta-analysis potentially allows for exploration of treatment effects and its interactions with individual patient characteristics.

In IPD meta-analysis, the individual-level data from each randomized clinical trial (RCT) is obtained, so IPD can be considered the original source material. There are several advantages of IPD meta-analysis. Firstly, IPD allows one to standardize analyses across studies and directly derive the information desired, independent of significance or how it was reported in the original study [17, 19]. Secondly, IPD may also include, more follow-up data, more participants, and more outcomes compared with aggregate meta-analysis as more data may be available to be pooled [17, 19]. Thirdly, additional analyses can be carried out to explore heterogeneity [17, 19]. Finally, a complete master database can be maintained for future collaborative initiatives as long as study authors are in agreement.

In addition to the many advantages of IPD, there are challenges to the use of IPD. One of the challenges of IPD is retrieving the datasets of all relevant RCTs. Not all datasets can be collected, because of several reasons. For example, authors, who published an RCT several years ago, may not be easily located, datasets may have been lost, authors are not willing to share their data, or authors are not allowed to share their data because of ethical reasons. In addition to collection of the data, the generation of a consistent data format across studies is very time consuming and may not be possible due to differences in measurement of domains across studies. Furthermore, IPD analyses require advanced statistical expertise.

Despite all these challenges, the advantages of using an IPD meta-analysis can be considerable both statistically and clinically compared to a meta-analysis of aggregate data which is why IPD meta-analyses are increasingly being applied [17].

On average, SMT for people with chronic LBP is moderately effective, SMT can relieve LBP in some patients but it does not seem to be effective for everyone. This differential response can be caused by moderators. Potential moderators, which have been identified in the literature, are gender, age, duration of back pain, psychosocial factors, and treatment preference or expectations [20]. In addition, in the literature there is little information about mediators that influence SMT for LBP. Aggregate meta-analysis is not well suited to examine mediation effects. This IPD may help to identify potential moderators and/or mediators of patients who improve or who fail to improve when treated with SMT, which can lead to predict improved outcomes and reduction in costs.

### Study objectives

The objectives of this study are as follows:

- To perform an IPD meta-analysis to assess the treatment effect of SMT compared to any other treatment for primary outcomes (pain and physical functioning) and secondary outcomes (perceived recovery, return-to-work or absenteeism, health-related quality of life, satisfaction with treatment, and reduction in frequency of analgesic use) at the short and long term follow-up periods in adults with chronic LBP.
- To explore potential SMT treatment effect moderation of individual patient characteristics measured at baseline according to prespecified theoretical framework (Tables 1 and 2). We will consider age, gender, duration of low back pain, psychosocial factors, and treatment preference/expectation) as candidate treatment effect moderators, while the exploratory moderators will only be used as a guide for future research (Tables 1 and 2).

**Table 1:** Overview of outcomes extracted for IDP analyses and the role of the variable in moderator analysis

Domain	Assessment instruments/measurements scales	Exploratory/confirmatory moderator analysis
Primary outcomes		
Pain	e.g., Visual Analog Scale Numerical Rating Scale, Aberdeen Back Pain Scale	Confirmatory (the baseline measurement of pain)
Physical functioning	e.g., Oswestry Disability Index, Roland Morris Disability Questionnaire	Confirmatory (the baseline measurement of physical functioning)
Secondary outcomes		
Recovery	Global assessment	
Return-to-work	e.g., numbers of days of work absenteeism, number of participants that returned to work	
Quality of life	Short Form-36 Item Health Survey, EuroQol (EQ5D)	Confirmatory (the baseline measurement of quality of life)
Satisfaction	Satisfaction with treatment Satisfaction with outcome	
Reduction in frequency of analgesic use

**Table 2:** Overview of prognostic factors extracted for IDP analyses and the role of the variable in moderator analysis

Individual subject characteristics	Prognostic factors	
	Age	Confirmatory
	Gender	Exploratory
	Height, weight, body mass index	Exploratory
	Ethnicity/race	Exploratory
Lifestyle factors		
	Participation in sports activities or physical fitness level	Exploratory
	Smoking	Exploratory
	Alcohol use	Exploratory
Sociodemographic characteristics		
	Marital status	Exploratory
	Level of education	Exploratory
	Income	Exploratory
	Employment status	Exploratory
Nature and severity of the low back pain		
	Duration of the low back pain	Confirmatory
	Non-specific	Exploratory
	Radiation	Exploratory
	Previous low back pain treatment received	Exploratory
Comorbidities, e.g., diabetes, heart disease		
	Presence of comorbidities	Exploratory
	Number of comorbidities	Exploratory
	Category of comorbidities	Exploratory
Type of treatment	Manipulation, mobilization, combination	
Psychosocial factors	e.g., Back Depression Inventory, Fear Avoidance Beliefs Questionnaire	Confirmatory
Treatment preference/ expectation	e.g., preference for treatment, previous experience with treatment, expectation to final improvement, self-efficacy scale/beliefs	Confirmatory
- To identify characteristics of the SMT interventions (e.g., manipulation/mobilization) that influence the treatment effect.
- To identify mediators of treatment effects (e.g., physical functioning may mediate the association between SMT and return-to-work). All mediators are exploratory and therefore only be used as a guide for future research.

## Methods

The protocol was developed according to the Preferred Reporting Items of Systematic Reviews and Meta-Analyses Protocol (PRISMA-P) guidelines [21], and the protocol has been registered on PROSPERO database (Ref:CRD42015025714). The PRISMA-P checklist is included as an additional file (see additional file 1).

### Criteria for including studies for this study

#### Types of studies and types of participants

Only RCTs will be included which evaluate the effects of SMT in adults (≥18 years of age) with an identifiable group of patient with chronic (≥12 weeks duration) non-specific LBP (alone or with leg pain) from primary or secondary care. Studies, which compare the effects of SMT as part of a multi-modal treatment, will be included as long as the effects of SMT can be determined, for example, SMT plus another intervention versus the same intervention alone. We will extract only the data on the patients with chronic LBP from RCTs with mixed acute and chronic LBP population, if feasible (we included only studies where the duration of LBP is more than 12 weeks of low-back pain in more than 50% of the population).

Studies using an inadequate randomization procedure (e.g., alternate allocation, allocation based on birth date) will be excluded as well as studies including individuals with LBP caused by specific pathologies (e.g., tumor, fracture). Studies including only patients with LBP and other conditions such as pregnancy or post-operative patients will also be excluded.

We will obtain only the studies published in 2000 or later, as we expect to receive more data from recent studies than older studies because it is difficult to trace authors of older studies, and there is a high probability that the data from older studies has been lost or destroyed. However, this is not likely to negatively influence our analysis because the quality of studies has improved over time and therefore, we expect that these newer studies will yield more valid answers to our questions (45)

### Types of interventions

#### Experimental intervention

The interventions examined in this review include both spinal manipulation and mobilization for chronic LBP. Unless otherwise indicated, SMT refers to both “hands-on” treatments.

#### Types of comparison

Studies will be included if the study design used suggests that the observed differences are due to the unique contribution of SMT. This excludes studies with a multi-modal treatment as one of the interventions (e.g., standard physician care + spinal manipulation + exercise therapy) and a different type of intervention or only one intervention from the multi-modal therapy as the comparison (e.g., standard physician care alone), thus rendering it impossible to decipher the effect of SMT. However, studies comparing SMT in addition to another intervention compared to that same intervention alone will be included. Comparison therapies will be combined into the following main clusters based on the Cochrane Review [4]:

1. SMT versus inert interventions
2. SMT versus sham SMT
3. SMT versus all other interventions
4. SMT in addition to any intervention versus that intervention alone

Inert interventions included, for example, detuned diathermy and detuned ultrasound. “All other interventions” included both presumed effective and ineffective interventions for treatment of chronic LBP. Determination of what interventions were considered ineffective and effective was based upon the literature and our interpretation of those results [4].

### Types of outcome measures

#### Primary outcomes

- Pain expressed on a self-reported scale (e.g., visual analog scale (VAS), numerical rating scale (NRS))
- Functional status expressed on a back-pain specific scale (e.g., Roland-Morris Disability Questionnaire, Oswestry Disability Index)

#### Secondary outcomes

- Health-related quality of life (e.g., SF-36 (as measured by the general health subscale), EuroQol, general health (e.g., as measured on a VAS scale) or similarly validated index)
- Return-to-work (self-reported or registry-based)
- Global improvement or perceived recovery (recovered is defined as the number of patients reported to be recovered or nearly recovered)
- Self-reported satisfaction with treatment
- Reduction in frequency of analgesic use (self-reported or registry-based)

## Search methods for identification of new studies

RCTs that were identified and described in the Cochrane Review on non-specific chronic LBP [4] will be used and complemented by additional RCTs which have been published later than the census date for the Cochrane Review.

All the steps, including the selection of studies and evaluation of the risk of bias will be conducted by two independent reviewers (SMR, AdeZ). Both review authors have a background in chiropractic and are practicing clinicians, but also have training in epidemiology. SMR is the principal author of the Cochrane Review of SMT for chronic LBP [4], which will ensure consistency in the evaluation of the risk of bias. When necessary, a third reviewer (RO) will be contacted.

### Electronic searches

The search strategy includes a computerized search of electronic databases since the last Cochrane Review update (June 2009 to December 2014) (Additional file 2):

- CENTRAL from The Cochrane Library 2009, issue 2 to December 2014
- MEDLINE from June 2009 to December 2014
- Embase from June 2009 to December 2014
- CINAHL from June 2009 to December 2014
- PEDro from June 2009 to December 2014
- Index to Chiropractic Literature from June 2009 to December 2014

The search will be in line with the recommendations of the Cochrane Back and Neck (CBN: formerly the Cochrane Back Review Group) which was used in Cochrane Review on non-specific chronic LBP. We will update the search mid-2016.

We will conduct citation searches of the previous review publications and screen cited references of other recent SMT systematic reviews [22, 23]. Also, we will identify abstracts which have been published after 2009 for which no full article has been published and search the trial register (ClinicalTrials.gov) for unpublished trials. Finally, we will contact content experts for additional trials.

## Data extraction of the additional RCTs

A standard protocol will be followed for study selection and data abstraction. Potentially relevant studies will be obtained in full text and independently assessed for inclusion. There will be no language restrictions.

We will extract data on study characteristics (e.g., country where the study was conducted, recruitment method, source of funding, patient characteristics (e.g., number of participants, age, gender), description of the experimental and control interventions, co-interventions, duration of follow-up, types of outcomes assessed, and the authors’ reported results and conclusions).

We will extract outcome data for all time periods reported in the original studies. We will define sufficiently similar categories of follow-up using the Cochrane Review as a guidance, which defined the following categories: 1, 3, 6, 12, and more than 12 months [4]. Outcomes will be categorized according to the time closest to these intervals.

### Assessment of risk of bias in included studies

We will use the assessment of the risk of bias already completed for the studies in the Cochrane Review. Risk of bias for each published study after June 2009 will be assessed using criteria recommended by the CBN [24]. These criteria are standard for evaluating effectiveness of interventions for LBP. The criteria will be scored as “yes,” “no,” or “unclear” and presented in the *Risk of Bias* table. Any disagreement will be resolved by discussion and the same independent third reviewer can be contacted if necessary.

### Collection of IPD

The original data will be sought from the authors of all studies fulfilling the inclusion criteria. The contact information of the authors of identified trials will be found in the publication, on PubMed or the internet. The authors will be sent an information about our IPD analysis by e-mail and will be asked to share their IPD along with their variable codebook. If no codebook is present, copies of their original data collection forms will be requested. If there is no response from the contact author other study authors will be contacted. Two reminders will be sent to all authors.

Previous contact by SMR for his Cochrane Review resulted in prompt assistance from most authors, particularly from recently published trials. Communication to date has resulted in 17 authors willing to participate. Several authors have published more than one trial.

Each participating study author will be sent an IPD policy form which contains information regarding data ownership, data confidentiality, data access and use, publication rules, and (co-) authorship. Authors will be asked to fill in an IPD data request form (this document asks for verification of eligibility criteria, the willingness to share information, and to provide contact details). Authors are required to sign the IPD Data Sharing Agreement (a contract between the author and the VU University describing the condition regarding data as stated in the IPD policy). Finally, the author receives an IPD Data Transfer Protocol (containing information on how to send the data). (see additional file 3)

Raw de-identified data will preferably be sent to the VU University Amsterdam by e-mail after the data are encrypted by a program such as Axcrypt; however, the methods for receiving raw data may vary depending on the security concerns from the participating institutions. Databases in all formats will be accepted. After the data have been received, they will be stored on a secure institutional server.

Data will be sought for all participants (this includes those who were excluded from the original analysis) at all time points and will be grouped later for analyses. We will collect and extract data in the domains described in the Tables 1 and 2.

### Preparing data for analyses

We will compare the original data with the published data to check for completeness and improbable values, and where possible, we will solve the discrepancies between our results and those presented in the original data, with the original study author.

**Fig. 1:**
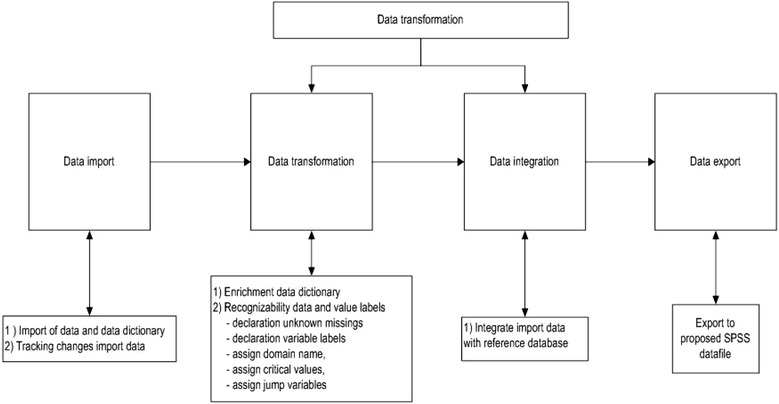
Data harmonization process. Reprinted with permission: Buffart LM, Kalter J, Chinapaw MJ, Heymans MW, Aaronson NK, Courneya KS, et al. Rationale and design for meta-analyses of individual patient data of randomized controlled trials that evaluate the effect of physical activity and psychosocial interventions on health-related quality of life in cancer survivors. Predicting OptimaL cAncer RehabIlitation and Supportive care (POLARIS): Systematic reviews. 2013;2:75

All variables will be harmonized in a data harmonization platform (DHP) developed for the POLARIS study [25]. Briefly, this DHP support us with the steps of importing and harmonizing the original studies with a master data dictionary and exporting the selected variables and studies into one harmonized SPSS dataset for the proposed statistical analyses (see Fig. 1). Our master data dictionary (see additional file 4) describes the data as extensively as possible allowing us to keep the original variable. Consequently, this leads to a gain in information for the analyses compared to aggregate data. For example, some studies may only report particular outcomes of the Oswestry Disability Index in their publications. In contrast, we can include all separate items from the Oswestry Disability Index in addition to the total score as well as including various measures for pain.

The DHP is used to rename, label, and integrate the variables for each included study with the master data dictionary in a consistent manner. If in doubt, we will contact primary study authors for clarification and/or discuss within the steering committee consisting of all authors.

Whenever possible, we will maintain data for continuous measurement of variables. If data on a variable of interest are not available in the dataset, we will attempt to extract this information based on other data in the set (e.g., sick leave variable is missing, but there is a variable on disability pension or workers compensation). We will address subject-level missing data on variables and outcomes if necessary. For example, missing baseline variable data will be handled using multiple imputation techniques, under a missing-at-random assumption, so as to avoid excluding patients from the analysis and to ensure that the baseline balance between treatment groups is maintained.

### Data analysis

#### Overall treatment effect of SMT in adults with LBP

We will perform IPD meta-analyses to assess the treatment effect of SMT for primary and secondary outcomes in adults with chronic LBP. The primary outcomes are pain- and back pain-related disability. The secondary outcomes of interest include perceived recovery, return-to-work or absenteeism, health-related quality of life, satisfaction with treatment, and reduction in frequency of analgesic use. In the first instance, we will pool data of different scales measuring the same construct. If, however, different scales measuring the same construct cannot be combined because the scales differ, the choice of the analysis will be determined by where the majority of the data lie.

If more than one measurement scale for a domain e.g., pain within one study has been collected, we will use the most common scale used within trials in the IPD database. If in a domain, different scales measure different constructs as in the case of functional status (e.g., Oswestry Disability Index, Roland Morris Disability Questionnaire) then this construct will be examined separately.

Comparison therapies will be combined into the following main clusters: (1) SMT versus inert interventions, (2) SMT versus sham SMT, (3) SMT versus all other interventions, and (4) SMT in addition to any intervention versus that intervention alone.

In the group, SMT versus all other interventions, we will look at the clinical homogeneity of the comparison. This may result in another classification, for example SMT versus exercise.

Our primary analyses will consist of one-stage IPD meta-analyses, taking into account within study clustering of study effects. These models will take the form of the following:

$$ {\gamma}_{i k}={\alpha}_i+{\beta}_i{x}_{i k}+{\theta}_i{z}_{i k}+{e}_{i k} $$

$$ {\theta}_i = \theta +{u}_i $$

$$ {u}_i\sim N\left(0,{\tau}^2\right) $$

$$ {e}_{ik}\sim N\left(0,{\sigma}^2\right) $$

where *γ*_*ik*_ refers to the estimated continuous outcome for the *k* ^th^ person in the *i* ^th^ study (for binary outcomes *γ*_*ik*_ refers to the logit of the outcome), *α*_*i*_ represents study-specific intercepts, *β*_*i*_ represents the adjustment for the baseline outcome, *u*_*i*_ is a random effect indicating the treatment effect in the *i* ^th^ trial, and *θ*_*i*_ is normally distributed around a pooled treatment effect $\mathbf{\mathsf{\boldsymbol{\uptheta}}}$ with between-study variance *τ* ^2^. *σ* ^2^ is the residual variance of the responses in trial *i* after accounting for the treatment effect.

In order to compare our results with the outcomes of the original studies and as a sensitivity analysis to our one-stage analyses, we will also conduct two-stage analyses which take the following form:

Stage 1: Model for each trial separately

$$ {\gamma}_{i k}={\alpha}_i+{\beta}_i{x}_{i k}+{\theta}_i{z}_{i k}+{e}_{i k} $$

$$ {e}_{ik}\sim N\left(0,{\sigma}^2\right) $$

Stage 2

The estimates of each trial $\left({\widehat{\theta}}_i\right)$ and the estimates of the variance V for each trial V $\left({\widehat{\theta}}_i\right)$ are subsequently pooled in a random effects model.

$$ {\widehat{\theta}}_i={\theta}_i+{\varepsilon}_i $$

$$ {\varepsilon}_i\sim N\left(0,\  V\left({\widehat{\theta}}_i\right)\right) $$

$$ {\theta}_i = \theta +{u}_i $$

$$ {u}_i\sim N\left(0,{\tau}^2\right) $$

The pooled treatment effect of SMT will be estimated according to a mean difference (for continuous outcomes) or an odds ratio (for binary outcomes) and their 95% CIs, based on the intention-to-treat (ITT) principle.

We recognize that variables may not be reported in all trials, and so, some analyses may need to be restricted to the subset of trials providing each variable of interest.

### Possible moderation of baseline characteristics on treatment response

We will examine treatment effect modification at the patient level, to assess whether individual patient characteristics measured at baseline are associated with treatment response.

Candidate moderators of treatment response have been identified (see Tables 1 and 2). The selection of these moderators was based on a specific rationale e.g., understanding behavioral and sociocultural mechanisms by which response is modified or from prognostic research (treatment effect modification studies or prognostic factor research) [20, 26, 27]. The interactions between the intervention and potential moderators will be examined.

Moderator analysis can be classified into confirmatory or exploratory. Moderators in confirmatory analyses are those related to specific theory or evidence, while moderators in exploratory analyses relate to moderators for which no empirical evidence exists or which a specific mechanism is lacking. These will be explored in order to inform future trials [28]. In Tables 1 and 2, we have indicated which analyses are confirmatory and which are exploratory [20, 26–32].

For the moderator analyses, we will extend the one-step IPD meta-analysis framework described above to include the potential effect modifiers and interaction terms between treatment and each variable. Potential moderators will be analyzed one by one in the following model.

$$ {\gamma}_{i k}={\alpha}_i+{\beta}_i{x}_{i k}+{\theta}_i{z}_{i k}+{\mu}_i{\omega}_{i k}+{\gamma}_w{z}_{i k}\left({\omega}_{i k}-{\overline{\omega}}_i\right) + {e}_{i k} $$

$$ {\theta}_i = \theta +{\gamma}_A{\overline{z}}_i\kern0.5em +{u}_i $$

$$ {u}_i\sim N\left(0,{\tau}^2\right) $$

$$ {e}_{ik}\sim N\left(0,{\sigma}^2\right) $$

where *μ*_*i*_ represents the patient-level covariate (fixed effect of the potential moderator), *γ*_*w*_ explains the patient-level variation in treatment response, *γ*_*A*_ represents the across trial interaction, and *τ* ^2^ represents the unexplained between-study variance.

If the interaction terms are statistically significant, we will present treatment effects for subgroups with the 95% confidence interval of size of the interaction terms. If convergence is achieved, we will investigate multiple moderators in the same analysis.We will discuss if those variables are clinically important effect modifiers in a consensus meeting. We will use the current knowledge on minimal clinically important changes within individual patients as guidance, because there is no current literature on minimal clinically important differences between groups. The current literature states that as any improvement in score ≥30% of its baseline value, with a minimum value of 20-point (/100) improvement in pain and 10-point (/100) improvement in functioning is clinically important [33, 34].

### Effects of characteristics of the intervention (e.g., manipulation/mobilization) on treatment response

We will use the one-stage IPD meta-analysis framework described above to assess the treatment effect of each type of SMT technique for primary in adults with chronic LBP, by stratifying the analyses by type of SMT.

Type of SMT technique groups will be manipulation, mobilization, or mixed where both techniques were used and will be compared to the same comparison groups as for the overall effect. These analyses will be exploratory as the mechanisms of how manipulation/mobilization works are not yet fully understood.

Where possible, for each analysis, we will compare the effect of SMT considering the dose (number of SMT treatments). We will recognize that this is a study-level comparison, and thus subject to potential study-level confounding

### Expected mediators of treatment effects

Mediators are causal links between the intervention and the outcome and identify how an intervention might achieve its effects. For example, physical functioning may mediate the association between SMT and return-to-work. Potential mediators of the intervention effect on the outcomes will be explored using the potential outcome framework [35–37].

All mediator analyses will be exploratory as there is little information in the literature on mediators that influence SMT for chronic LBP. Mediators in the treatment of LBP that have been identified are mostly cognitive and psychological mediators like sleep, fear beliefs, self-efficacy, stress, and satisfaction [38]. The effects of these mediators will be explored as well as the mediator pain for physical function and return-to-work.

### Sensitivity analyses

In order to determine the robustness of the main analyses, sensitivity analyses will be conducted to assess the impact of our review methods, decisions, and definitions.

An analysis will be carried out to assess the effects of imputing missing data by comparing several imputation methods [17, 39, 40]. We will perform sensitivity analyses in which data from persons with missing baseline values will be imputed following the two-stage model proposed by Burgess et al [41]. In this model, data are first imputed within studies and treatment effects are derived using Rubin’s Rules and subsequently, these effect estimates are pooled by inverse variance weighting [41]. In addition, inclusion of only studies with a low risk of bias, where studies with a low risk of bias will be defined as those that fulfill six or more of the 12-criteria items, will be performed to assess the impact of studies of lower methodological quality on the findings. Besides dividing the studies in a low and high risk of bias, we will compare studies with difference in the criteria of the risk of bias (for example, concealment versus no concealment of treatment allocation).

Lastly, additional sensitivity analyses will be performed related to the definitions of sufficiently similar measures for patient-level variables and the definitions of follow-up time points. Not all studies have the same follow-up evaluations of the outcomes. Data might either be measured at different time points or come from different outcome measures. In addition, in many of the included studies, the potential moderators or moderators might not be available. This will limit us in the possible analyses.

### Publication policy

A Back Pain IPD Consortium was formed consisting of a steering committee, an international advisory committee and collaborators. The steering committee will be responsible for the daily management of the study and its coordination. The international advisory committee consists of experts in the field of LBP and SMT and therefore, is in the position to give specific advice on SMT as it relates to the field of LBP. Collaborators are the principle investigators of a RCT. We will invite new collaborators as new eligible studies become available.

A meeting for collaborators and international advisory committee will be held to update the members on the progress of the study and to discuss the challenges encountered.

The primary publication of the results of this review will be prepared by the steering committee. These results will be circulated to the members of the Back Pain IPD Consortium for a critical comment. The collaborators and international advisory committee members will be listed as the Back Pain IPD group, and all participating investigators contributing to this project will be listed at the end of each publication. All co-authors need to comply with the criteria of the Vancouver Protocol for co-authorship.

In addition to the present analysis, we intend to establish a repository for future use of these data.

## Discussion

In this project, we will perform an IPD meta-analysis on SMT for chronic LBP. We aim to examine the main effects of SMT on primary and secondary outcomes, as well as to analyze possible moderator and mediator effects. In addition, we will examine whether there are differences in outcome based upon different types of SMT. The results of our analyses will be compared to the results from the previously conducted aggregate meta-analyses [4]. Studies have shown that IPD meta-analysis due to the increased sample size, consistent presentation of data, and additional analysis to explore heterogeneity can be more reliable and valid than aggregate data meta-analysis [19], although this difference might also be attributed to the number of studies that can be used or the amount of data that can be utilized.

Therefore, this project may identify important gaps in our knowledge with respect to prognostic factors of treatment effects. Besides that, this project will help to improve quality, design, and reporting of LBP trials with respect to collection of information on prognostic factors relevant to the identification of treatment subgroups .

Finally, one of the loftier goals of this IPD study is to establish an international collaborative group on IPD in the field of LBP. This will provide in the future a unique opportunity to compare the effect of different treatment modalities and to investigate gaps in the literature, including comparison of the results of traditional meta-analysis using standard aggregate-level approaches, multi-treatment meta-regression, and IPD.

## Additional files

Additional file 1: PRISMA-P (Preferred Reporting Items for Systematic reviews and Meta-Analyses Protocols) 2015 checklist: recommended items to address in a systematic review (DOC 84 kb)
Additional file 2: Search strategy (DOCX 27 kb)
Additional file 3: IPD policy form including IDP sharing agreement, IPD data request form, and IPD transfer protocol (DOCX 252 kb)
Additional file 4: Codebook (XLSX 203 kb)

## Abbreviations

DHP: Data harmonization platform
IPD: Individual participant data
LBP: Low back pain
PRISMA-P: Preferred Reporting Items of Systematic Reviews and Meta-Analyses Protocol
RCT: Randomized clinical trial
SMT: Spinal manipulative therapy
