# Supplementary material for: Rational and design of an individual participant data meta-analysis of spinal manipulative therapy for chronic low back pain—a protocol
Source: Syst Rev. 2017 Jan 26;6:21. doi: 10.1186/s13643-017-0413-y (PMC5267437; doi:10.1186/s13643-017-0413-y)
Supplement: Additional file 2: — Search strategy (DOCX 27 kb) [file 13643_2017_413_MOESM2_ESM.docx]

**Additional file 2 Search Strategy**

1. #1 MeSH descriptor Back explode all trees
2. #2 MeSH descriptor Buttocks, this term only
3. #3 MeSH descriptor Leg, this term only
4. #4 MeSH descriptor Back Pain explode tree 1
5. #5 MeSH descriptor Back Injuries explode all trees
6. #6 MeSH descriptor Low Back Pain, this term only
7. #7 MeSH descriptor Sciatica, this term only
8. #8 (low next back next pain)

9. #9 (lbp)

1. #10 (#1 OR #2 OR #3 OR #4 OR #5 OR #6 OR #7 OR #8 OR #9)
2. #11 MeSH descriptor Musculoskeletal Manipulations explode all trees
3. #12 MeSH descriptor Chiropractic explode all trees
4. #13 manip*
5. #14 MeSH descriptor Osteopathic Medicine explode all trees
6. #15 osteopath*
7. #16 chiropract*
8. #17 (#11 OR #12 OR #13 OR #14 OR #15 OR #16)
9. #18 (#17 AND #10)
10. #19 (#18)

**Appendix 2. MEDLINE Search Strategy**

1. Clinical Trial.pt.

2. randomized.ab,ti.

3. placebo.ab,ti.

4. dt.fs.

5. randomly.ab,ti.

6. trial.ab,ti.

7. groups.ab,ti.

8. or/1-7

9. Animals/

10. Humans/

11. 9 not (9 and 10)

12. 8 not 11

13. dorsalgia.ti,ab.

14. exp Back Pain/

15. backache.ti,ab.

16. (lumbar adj pain).ti,ab.

17. coccyx.ti,ab.

18. coccydynia.ti,ab.

19. sciatica.ti,ab.

20. sciatica/

21. spondylosis.ti,ab.

22. lumbago.ti,ab.

23. exp low back pain/

24. or/13-23

25. exp Manipulation, Chiropractic/

26. exp Manipulation, Orthopedic/

27. exp Manipulation, Osteopathic/

28. exp Manipulation, Spinal/

29. exp Musculoskeletal Manipulations/

30. exp Chiropractic/

31. manipulation.mp.

32. manipulate.mp.

33. exp Orthopedics/

34. exp Osteopathic Medicine/

35. or/25-34

36. 12 and 24 and 35

37. limit 36 to yr=”2007 - 2008“

**Appendix 3. EMBASE Search Strategy**

|  | |  |
| --- | --- | --- |
| 1. | Clinical Article/ |  |
| 2. | exp Clinical Study/ |  |
| 3. | Clinical Trial/ |  |
| 4. | Controlled Study/ |  |
| 5. | Randomized Controlled Trial/ |  |
| 6. | Major Clinical Study/ |  |
| 7. | Double Blind Procedure/ |  |
| 8. | Multicenter Study/ |  |
| 9. | Single Blind Procedure/ |  |
|  | |  |
| 10. | Phase 3 Clinical Trial/ |  |
| 11. | Phase 4 Clinical Trial/ |  |
| 12. | crossover procedure/ |  |
| 13. | placebo/ |  |
| 14. | or/1-13 |  |
| 15. | allocat$.mp. |  |
| 16. | assign$.mp. |  |
| 17. | blind$.mp. |  |
| 18. | (clinic$ adj25 (study or trial)).mp. |  |
| 19. | compar$.mp. |  |
| 20. | control$.mp. |  |
| 21. | cross?over.mp. |  |
| 22. | factorial$.mp. |  |
| 23. | follow?up.mp. |  |
| 24. | placebo$.mp. |  |
| 25. | prospectiv$.mp. |  |
| 26. | random$.mp. |  |
| 27. | ((singl$ or doubl$ or trebl$ or tripl$) adj25 (blind$ or mask$)).mp. |  |
| 28. | trial.mp. |  |
| 29. | (versus or vs).mp. |  |
| 30. | or/15-29 |  |
| 31. | 14 and 30 |  |
| 32. | human/ |  |
| 33. | Nonhuman/ |  |
| 34. | exp ANIMAL/ |  |
| 35. | Animal Experiment/ |  |
| 36. | 33 or 34 or 35 |  |
| 37. | 32 not 36 |  |
| 38. | 31 not 36 |  |
| 39. | 37 and 38 |  |
| 40. | 38 or 39 |  |
| 41. | dorsalgia.mp. |  |
| 42. | back pain.mp. |  |
| 43. | exp BACKACHE/ |  |
| 44. | (lumbar adj pain).mp. |  |
| 45. | coccyx.mp. |  |
| 46. | coccydynia.mp. |  |
| 47. | sciatica.mp. |  |
| 48. | exp ISCHIALGIA/ |  |
| 49. | spondylosis.mp. |  |
| 50. | lumbago.mp. |  |
| 51. | exp Low back pain/ |  |
| 52. | or/41-51 |  |
| 53. | exp CHIROPRACTIC/ |  |
| 54. | exp Orthopedic Manipulation/ |  |
| 55. | exp Manipulative Medicine/ |  |
| 56. | exp Osteopathic Medicine/ |  |
| 57. | manipulation.mp. |  |
| 58. | manipulate.mp. |  |
| 59. | exp Orthopedics/ |  |
| 60. | osteopathy.mp. |  |
| 61. | or/53-60 |  |
| 62. | 40 and 52 and 61 |  |
|  | |  |

**Appendix 4. CINAHL Search Strategy**

Yields 44 for 2007-2008

1. Randomized Controlled Trials.mp.
2. clinical trial.pt.
3. exp Clinical Trials/
4. (clin$ adj25 trial$).tw.
5. ((singl$ or doubl$ or trebl$ or tripl$) adj25 (blind$ or mask$)).tw.
6. exp PLACEBOS/
7. placebo$.tw.
8. random$.tw.
9. exp Study Design/
10. (latin adj square).tw.
11. exp Comparative Studies/
12. exp Evaluation Research/
13. Follow-Up Studies.mp.
14. exp Prospective Studies/
15. (control$ or prospectiv$ or volunteer$).tw.
16. Animals/
17. or/1-15
18. 17 not 16
19. dorsalgia.ti,ab.
20. exp Back Pain/
21. backache.ti,ab.
22. (lumbar adj pain).ti,ab.
23. coccyx.ti,ab.
24. coccydynia.ti,ab.
25. sciatica.ti,ab.
26. exp SCIATICA/
27. spondylosis.ti,ab.
28. lumbago.ti,ab.
29. exp low back pain/
30. or/19-29
31. exp CHIROPRACTIC/
32. exp MANIPULATION, CHIROPRACTIC/
33. exp MANIPULATION, ORTHOPEDIC/
34. exp MANIPULATION, OSTEOPATHIC/
35. manipulation.mp.
36. manipulate.mp.
37. exp Manual Therapy/
38. exp ORTHOPEDICS/
39. exp OSTEOPATHY/
40. or/31-39
41. 18 and 30 and 40
