# Supplementary material for: Rational and design of an individual participant data meta-analysis of spinal manipulative therapy for chronic low back pain—a protocol
Source: Syst Rev. 2017 Jan 26;6:21. doi: 10.1186/s13643-017-0413-y (PMC5267437; doi:10.1186/s13643-017-0413-y)
Supplement: Additional file 3: — IPD policy form including IDP sharing agreement, IPD data request form, and IPD transfer protocol (DOCX 252 kb) [file 13643_2017_413_MOESM3_ESM.docx]

# **Back Pain IPD Policy Document**

## ****Rationale****

Low back pain (LBP) is a leading cause of pain and disability and as a result has a major socioeconomic impact. The majority of the cost associated with LBP is generated by those patients who have persistent chronic LBP. For LBP spinal manipulative therapy (SMT) is one of several interventions which evidence suggests is moderately effective. In 2011, a Cochrane review was conducted which investigated the effectiveness of SMT for chronic low back pain. However, one of the problems with traditional meta-analyses is the use of aggregate (mean) data while the use of individual patient data (IPD) allows for a better interpretation of the effects in select subgroups.

## **Aims**

- To assess the treatment effect of SMT for reducing pain and disability in adults with chronic LBP patients with IPD.
- To identify subgroups of patients with LBP who are more likely to benefit from SMT.
- To identify subgroups of characteristics of SMT which influence the treatment effect such as design and delivery characteristics (e.g. practitioner: chiropractor, manual therapist or other therapist) and types of manipulative techniques (mobilization, manipulation).

Funding

The IPD study is supported by a grant of the European Chiropractic Union.

## Back Pain IPD Consortium

The Back Pain IPD Consortium consists of the steering committee, the international advisory committee and all collaborators.

## Steering Committee

Coordination of the IPD study is performed at the Department of Health Science and the EMGO+ Institute for Health and Care Research, VU University Amsterdam. The Steering Committee is responsible for the study coordination, and consists of: Prof. M.W. van Tulder, Prof. R.W.J.G. Ostelo, Dr. S.M. Rubinstein, Dr. M.R de Boer and A. de Zoete (VU University Amsterdam) and Dr. J. Hayden (Dalhousie University, Halifax, Canada).

## International Advisory Committee

The International Advisory Board is established to give the project team advice about different issues in the field of low Back Pain and spinal manipulative therapy. The International Advisory Board consists of: Prof. M. Underwood, Prof. N.E. Foster, Prof. G Bronfort, Prof. C. Maher and Prof. J. Hartvigsen.

**Collaborators**

Collaborators are principle investigators of a research group providing data from the RCT for the IPD database.

## Definitions

*Recipient*: A person that uses the data from the Back Pain IPD database for proposed analyses.

*Third parties*: All parties (e.g., researchers) that are not members of the Back Pain IPD consortium.

## Data ownership and data confidentiality

- *Collaborators* have to sign the Data Sharing Agreement before transferring their data to the Back Pain IPD database.
- *Collaborators* have to confirm that they are authorized to provide the data to the Back Pain IPD database.
- The data made available for the Back Pain IPD database are and remain the property of the *Collaborator.*
- All data that are included in the Back Pain IPD database will be stored securely at the VU Amsterdam and are treated as confidential.
- All data in the Back Pain IPD database are anonymous; all confidential and privacy sensitive information is removed, and the data are not traceable to patients.
- *Collaborators* may decline participation on a paper-by-paper basis, without giving any reason.

## Data access and use

- Data from the Back Pain IPD database will be used by the Steering Committee (or another *recipient*) under the rules provided in this document.
- The data in the Back Pain IPD database are only to be used for the proposed analyses, and are not allowed to be used for other studies nor should they be provided to third parties without written permission from each *Collaborator*. The *recipient* will destroy the Data after being analyzed for the proposed study and send the syntax file(s) of the data analysis to VU University where it will be stored securely for backup purposes.
- Back Pain IPD data are only available for non-commercial scientific research.

# (co-) Authorship

- The Back Pain IPD Consortium complies with the Vancouver Protocol, i.e. authorship credit should be based on (1) substantial contributions to conception and design, or acquisition of data, or analysis and interpretation of data; (2) drafting the article or revising it critically for important intellectual content; and (3) final approval of the version to be published.
- Authors are recipients who have designed the study, have conducted the analysis and have written the paper.
- In all publications, the Back Pain IPD Consortium will be mentioned as co-author, i.e. ‘(on behalf of) the Back Pain IPD Consortium’; all collaborators in a specific analysis will be member of the consortium for the paper on that analysis.
- The participating studies and investigators of the Back Pain IPD Consortium will be listed at the end of each publication in the acknowledgements, with a maximum of 2 investigators per study (Principal Investigator and co-Principal Investigator).
- Names of leading authors conducting the analysis and writing the paper will be listed separately in addition to the Back Pain IPD Consortium.

# Information and communication

- All questions regarding the IPD study can be e-mailed to a.de.zoete@vu.nl or asked by telephone at +31 20 5982545.

**Back Pain IPD Data Sharing Agreement-Contributor**

R**esearch Project Title:** Use of IPD meta-analysis to inform effective management of spinal manipulative therapy for patients with chronic low Back Pain.

This Agreement is entered into on [date]

BETWEEN:

This Data Sharing Agreement is drawn up between:

VU University Amsterdam

**Department of Health Sciences, Faculty of Earth & Life Sciences, VU University, De Boelelaan 1085, 1081 HV Amsterdam**, **The Netherlands** and duly represented for the purpose hereof by prof.dr. M.W.van Tulder, principal investigator and

**…………………………………(Insert Name Collaborator)** (referred to as ***the Collaborator*** hereafter)

WHEREAS a project team, established to coordinate the Back Pain IPD database, has drafted the Back Pain IPD policy document, which is attached to this agreement as Annex I.

WHEREAS the *Collaborator* has agreed to provide data to be used by (a) third party/ies (referred to as ***the Recipient(s)*** hereafter*)* and stored in the Back Pain IPD database by the VU University.

This agreement commences on [INSERT DATE] and will terminate on [INSERT DATE] or upon completion of the Study, whatever occurs later, and unless extended and/or terminated by mutual agreement of both parties in writing, at which point in the event of extension an Amendment will be issued by the VU University to replace this document.

**1. Definitions**

**“Anonymous Data”** mean data that have been provided by the Collaborator in such a way that the information concerning personal or material circumstances can be attributed to an identified or identifiable individual only with a disproportionate amount of time, expense and/or labour, with the effect that this data is to be seen as being anonymous in a legal sense and as such is provided as coded and can NOT be traced back/relinked to an individual patient, but solely by the Collaborator.

**“Informed Consent”.** The written, signed and dated consent from the Subject or its legal representative, based on sufficient and understandable information, covering the collection, storage and use of its Personal Data.

## “Permitted Use”. The purpose for which the Data are used under this Agreement which purpose is limited to that described in the Back Pain IDP Policy Document attached hereto, and in the summary of the Study.

**“Study”.** Study means the study the Recipient wishes to conduct and for which she needs the Data, and more specifically investigate the epidemiology, natural history and treatment outcomes of Lower Back Pain (LBP) spinal manipulative therapy (SMT) and its moderately effectivity.

## “Subject”. The individual from whom the Personal Data originated or to whom the Data are related to.

**2. Description of Data to Be Provided**

Under the terms and Conditions provided herein, Collaborator, __________________________________________ will provide the following data: Individual Anonymous Data with descriptions of variable coding **AND/OR** scored variable databases with descriptions of variable coding (referred to as “the **Data**” hereafter) and if available data on economical evaluation to VU University and at the VU University the Data will be prepared for storage in the Back Pain IPD database owned by the VU University. The Data will come from completed randomised controlled trials.

The Collaborator is fully responsible for providing only Anonymous Data and the VU University of Amsterdam will treat it as such, hence it is not the responsibility of the VU University of Amsterdam if undesired disclosure of personal Data may occur due to the recipient from the Collaborator of Subject’s personal data, but agrees to use reasonable efforts to inform the Collaborator of such undesired disclosure, so that the Collaborator will take any measures he/she considers appropriate to correct the situation.

## The Collaborator represents that it has obtained Informed Consent from each Subject in accordance with its Applicable Law, which Informed Consent allows for the transfer of the Data for the Permitted Use.

**3. Access**

3.1 The Collaborator agrees to the access policy as set out in the Back Pain IPD policy document attached hereto.

**4. Use of Data**

4.1 The Recipient shall agree that the Data are solely used by the Recipient for the implementation of the Study. The Recipient will not assign, transfer, sell or otherwise disclose the Data to any third party. The Recipient will destroy the Data after being analysed for the proposed study and send the syntax file(s) of the data analysis to the VU University where it will be stored securely for backup purposes.

4.2 The Recipient shall agree to store the Data securely and not to provide it to any other party without the explicit written consent of Collaborator.

4.3 Access to the repository is restricted to those named in Table 1 of this agreement. Any changes will be notified in writing to [INSERT INSTITUTION NAME].

***Table 1 - Individuals who will have access to and use of the repository***

| **Permitted Users** | **Job title – Organization they work for – Where they will access data** |
| --- | --- |
| Annemarie de Zoete | Junior Researcher - VU University – VU University Amsterdam |
| Michiel de Boer | Assistant Professor - VU University – VU University Amsterdam |
| Joeri Kalter | Junior Researcher - VU medical center - VU medical center Amsterdam |

**5. Warranties, Indemnification and Limitation of Liability**

5.1 All Data supplied to the VU University are, to the Collaborator ’s reasonable knowledge and belief, true and accurate.

5.2 All Data that are included in the Back Pain IPD database shall be stored securely at the VU University and are treated as confidential.

5.3 The Collaborator will indemnify the VU University and hold the VU University harmless in respect of any loss, claims, damage or liability, including third party claims, of whatsoever kind or nature, which may arise out of or in connection with the use, handling, storage or disposal of the Data by the Recipient and/or the Subjects

5.4 In no event shall the VU University be liable to the Collaborator or any third party for any special, incidental, consequential, exemplary, punitive, direct or indirect damages including, without limitation, loss of goodwill, loss of profits or revenue, loss of savings, work stoppage or data loss arising out of or in any manner connected with this agreement.

**6 Publication**

6.1 In all publications, the Back Pain IPD Consortium will be mentioned as co-author, i.e. ‘ (on behalf of) the Back Pain IPD Consortium’; all collaborators in a specific analysis will be member of the consortium for the paper on that analysis.

However, each Collaborator may decide to withdraw his/her co-authorship at his/her discretion.

Collaborator shall receive the manuscript 15 days prior to its submission for review and comments, provided that scientific content shall be at the control of the VU University Amsterdam. Control and comments will be considered and incorporated in good faith. In the event that the Collaborator does not provide comments to the prospective publication within the period provided herein it is understood that he/she agrees with the publication and shall be included as author when appropriate in accordance with the standards generally accepted by the scientific community, including limited to the Vancouver Protocol. Provided that in all events, the Collaborator’s Data contribution shall be acknowledged in any publication related thereto.

**7. Intellectual Property**

7.1 Any and all intellectual property rights in and to the Data are and remain the sole and exclusive property of the Collaborator.

7.2 The VU University Amsterdam shall have the right to protect intellectual property (‘IP’) rights derived from using the Data, but must be notified into the Collaborator and it is agreed by both Parties that the Collaborator retains at all times the right to use the results and IP resulting from the Study for its own scientific non-commercial research and educational purposes.

**8. Law and Jurisdiction**

8.1 This agreement is governed by Dutch law.

8.2 The courts of Amsterdam shall have exclusive jurisdiction in relation to any dispute concerning this agreement.

**9. Term and Termination**

9.1 This agreement shall commence on the date set out above and shall continue until it expires or it is terminated in accordance with its terms.

9.2 This agreement may be modified and amended in writing by mutual consent of the Collaborator and the VU University.

9.3 If any part or any clause of this agreement proves to any extent invalid or unenforceable in law, the remainder of such clause and all other clauses of this agreement shall remain valid and enforceable to the fullest extent permissible by law, and such clause shall be deemed to be omitted from this agreement to the extent of such invalidity or unenforceability.

9.4 The VU University and the Collaborator may terminate this agreement, upon written notice to the other, if the other party materially breaches any term or provision of this agreement and fails to cure that breach within 6 weeks after receiving written notice thereof from the other party.

9.5 The terms of this agreement that by their nature should survive the termination of this agreement shall so survive, including, without limitation, clauses 4, 5, 6 and 7.

Agreed and signed in duplicate,

_____________________________ _____________________________

(signature) (signature)

_____________________________ _____________________________

Authorized Representative Authorized Representative

VU University Amsterdam

(name and function) (name and function)

Read and Understood,

_____________________________ _____________________________

(signature) (signature)

_____________________________ _____________________________

Prof. dr. M.W. van TUlder

Principal Investigator Principal Investigator

(name and function) (name and function)

_____________________________ ______________________________

(date) (date)

**Data Request Form**

We would like to thank you for participating in our individual patient data meta-analysis on spinal manipulation for low back pain. This data request form is used to collect information on details of your trial. Please fill in the form as completely as possible. We kindly request you to return the Data Request Form by using the e-mail submission button below. This submission button directs you to your e-mail server and places the Data Request Form as an attachment.

Furthermore, we kindly request you to e-mail the signed Data Sharing Agreement.

For any questions or support please contact Annemarie de Zoete. **Tel**: +31 20 5982545 or **E-mail:** [a.de.zoete@vu.nl](mailto:a.de.zoete@vu.nl)

Name of trial:

Study acronym:

Are you willing to share the raw dataset for the IPD study?

Are you willing to share the raw dataset for the International Low Back pain database?

First corresponding Author

Title: choose item.

Last Name:

First Name:

Initials:

Address:

City: Postal code:

Country: Email: Telephone: Skype account:

Second corresponding Author

Title: choose item.

Last Name:

First Name:

Initials: Address:

City:

Postal code: Country: Email: Telephone: Skype account:

**Trial Design**

Was the trial approved by the Medical Ethical Committee?

Was the protocol registered?

If yes, please provide your trial/protocol number (e.g. ISRCTN, NCT):

Was informed consent obtained from each patient?

How many arms were included in the study?

Type of intervention:

Please give details of other invention(s)

Was a sample size calculation performed?

Number of patient allocated to the randomized SMT group:

Number of patient allocated to the randomized control group 1: randomized control group 2:

randomized control group 3:

**Missing data**

Was a method used to handle missing data ?

If yes, what method was used (e.g., last observation carried forward, multiple imputation)?

Were the primary analyses based on intention-to-treat or per protocol analysis?

**Data transfer**

Please provide complete raw data on all patients randomized and if available all raw data on the economic evaluation. Data can be supplied in almost any format (SPSS, STATA, SAS, etc.). Please indicate which format has been used. Data files should be encrypted for which for example AxCrypt can be used. ***Encrypting*** your ***data*** makes it completely unreadable to anyone but you or its intended recipient.

In which type of format do you prefer to send the data? e.g. SPSS

Are you willing to provide your data by e-mail?

*If no*, please provide details about the way you prefer to provide your data (for example, via the cloud or send on a USB stick.

All data has to be recoded in a uniform format before entering in the IPD database.

Do you prefer the IPD Project team to recode your data according to the codebook of the IPD database or would you like to do this for yourself?

Which method of data encryption do you prefer? *(e.g. AxCrypt, Veracrypt)*

# Appendix 8 : IPD Data Transfer Protocol

**IPD Data Transfer Protocol**

This document contains a roadmap how to use Axcrypt for encryption. If you want to use another encryption program, please let us know which kind of program you want to use.

For any questions, you can contact me by telephone (+31 20 44) or by e-mail at [a.de.zoete@vu.nl](mailto:a.de.zoete@vu.nl).

**AxCrypt encryption method**

If you don’t have AxCrypt yet, you can download Axcrypt for free at:

<http://sourceforge.net/projects/axcrypt/files/AxCrypt/1.7.2867/AxCrypt-1.7.2867.0-Setup.exe/download>

*Step 1*: Download and install **AxCrypt** on your computer.

*Step 2*: Right click on the data file you want to send to IPD


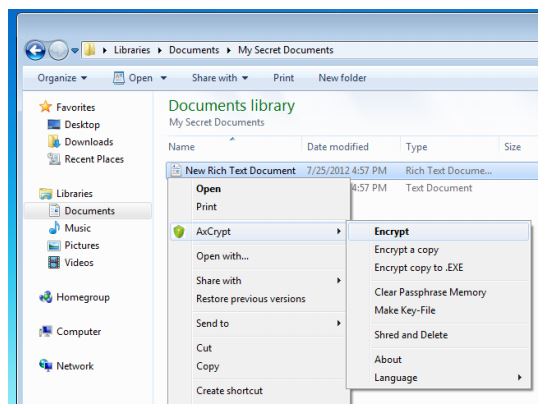


*Step 3*: This right-click menu is called the context menu. This is where you’ll find most of the

functionality of **AxCrypt**. Chose *language* (if language is not appropriate), otherwise select ‘**Encrypt’**.


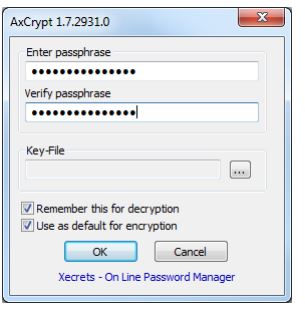


*Step 4*: You’ll be asked to provide a **pass phrase** and optionally a key-file.

*Step 5*: Enter a **pass phrase**, i.e. a sequence of secret strings. This is the secret that will protect your data from viewing by others and undetected tampering

*Step 6*: Enter your **pass phrase** a *second time* for verification. It’s vital that you ensure that you actually type what you think you type, and that you remember this pass phrase.

*Step 7*: Click **‘OK’**

*Step 8*: The file in encrypted. You can send the encrypted file and the pass phrase in *two separate e-mails* to [a.de.zoete@vu.nl](mailto:a.de.zoete@vu.nl)

**Thank you for your cooperation!**
